# A Deep Learning Approach to Intrusion Detection and Segmentation in Pellet Fuels Using Microscopic Images

**DOI:** 10.3390/s23146488

**Published:** 2023-07-18

**Authors:** Sebastian Iwaszenko, Marta Szymańska, Leokadia Róg

**Affiliations:** 1 Department of Acoustics, Electronics and IT Solutions, GIG Research Institute, 40-166 Katowice, Poland; 2 Department of Solid Fuels Quality Assessment, GIG Research Institute, 40-166 Katowice, Polandlrog@gig.eu (L.R.)

**Keywords:** deep learning, computer vision, image analysis, microscopy, petrography, intrusion detection

## Abstract

Pellet fuels are nowadays commonly used as a heat source for food preparation. Unfortunately, they may contain intrusions which might be harmful for humans and the environment. The intrusions can be identified precisely using immersed microscopy analysis. The aim of this study is to investigate the possibility of autonomous identification of selected classes of intrusions using relatively simple deep learning models. The semantic segmentation was chosen as a method for impurity identification in the microscopic image. Three architectures of deep networks based on UNet architecture were examined. The networks contained the same depth as UNet but with a successively limited number of filters. The input image influence on the segmentation results was also examined. The efficiency of the network was assessed using the intersection over union index. The results showed an easily observable impact of the filter used on segmentation efficiency. The influence of the input image resolution is not so clear, and even the lowest (256 × 256 pixels) resolution used gave satisfactory results. The biggest (but still smaller than originally proposed UNet) network yielded segmentation quality good enough for practical applications. The simpler one was also applicable, although the quality of the segmentation decreased considerably. The simplest network gave poor results and is not suitable in applications. The two proposed networks can be used as a support for domain experts in practical applications.

## 1. Introduction

The increased awareness of the impact of solid fuels on the environment and especially on climate change has led to investigations in the field for possible low emission replacements. One such surrogate is the fuel for barbecues (food preparation market) and wood pellets (used in the power industry). The markets for both of these solid fuel alternatives are steadily increasing. Both are considered renewable energy sources because of their composition on biomass—the released carbon dioxide was formerly up taken by the plants used for their production. Despite the use of the mentioned fuels for energy production, there is a lack of information on their quality. Needless to say, they may be a major threat to the environment and to humans. The widespread use of these fuels for the thermal treatment of food should be safe and reasonably harmless, and these conditions can only be met by uncontaminated fuel of the highest quality. It is therefore necessary to examine the purity of the solid biomass of fuels available on the market and to examine whether or not they are of the highest quality before reaching consumers.

The use of fuels for grilling is becoming increasingly popular. Users can choose from a wide range of pellets specifically produced for grilling. As the consumption of these fuels increases, doubts arise about their quality and how to control and ensure it.

The quality of grilling fuels should be of paramount importance because the smoke generated from their combustion comes into direct contact with food and significantly impacts human health and the environment. Smoke from wood pellets can contain hundreds of harmful pollutants, including carcinogenic and mutagenic compounds [1,2,3], heterocyclic amines [3,4], and polycyclic aromatic hydrocarbons [5,6,7,8]. These compounds are hazardous to human health and can lead to bronchitis, emphysema, or respiratory tract cancer.

Contamination of wood pellets with oils, paints, adhesives, and other organic compounds [9,10,11] is also dangerous to humans and the environment. Mineral material contamination may come from the wood harvesting process, while metallic additives and rust can enter the pellets during the production process. Petroleum products, adhesives, and treated wood are often added to increase the energy output and facilitate ignition [12].

Currently, there are standards and certification methods concerning the quality of wood pellets, but they are only used to assess the quality of wood pellets used for heating purposes. There are no dedicated standards for evaluating the quality of wood pellets intended for food processing [13].

While some solid biomass fuels are rigorously tested, others are tested occasionally or not at all [10,12,13]. For charcoal and charcoal briquettes, the standard has been developed [14], specifying requirements for quality and, more specifically, for a reduction in the amount of solid impurities. According to this standard, the determination of these impurities is carried out using the microscopic method. The requirements of this standard, in the microscopic part, can also be applied to the other two grill fuels mentioned above and to wood pellets used in the energy sector. Despite the introduction of the standard, quality tests of barbecue fuels and wood pellets are not used because of the difficulties of the process. Moreover, there are very few companies that could undertake such tests.

Optical microscopy is an effective tool for assessing the quality of wood pellets and is a very valuable supplement to physical and chemical tests recommended by applicable standards. It allows for the indisputable identification of individual contaminants. Microscopic identification of contaminants in wood pellets is a relatively new field of study. Its foundations were developed during thorough and very labor-intensive research, consisting of the development of the method for embedding organic material from wood pellets into the appropriate organic resins. This enables polishing the samples to a mirror-like surface suitable for microscopic examination in immersion. The following stage of the research was the identification of individual impurities and all their forms using the microscopic image [12]. This stage is usually also very tedious and time consuming.

Currently, microscopic analysis is successfully used to identify a wide range of contaminants in various grill fuels and wood pellets [10,12,15,16]. However, the process is laborious and time-consuming. The operator is required to have significant experience in recognizing these contaminants. This has inspired research in the application of deep learning in this area. The positive effects of using these methods in hard coal petrography [17,18,19] and also in geology [20,21] are already known.

The analysis of impurities in barbecue fuels and wood pellets is highly absorbent and time-consuming. It requires detection of many solid impurities, such as bark, fossil coals, petroleum substances, coke, plastics, glass, slag, rust, metals, mineral matter, and others. To the authors best knowledge, the analysis has not been automated yet. It requires deep competence and experience from the operator. The purpose of this study is twofold. First, it is an attempt to propose a way of automating this process, based on the application of computer vision methods and tools to the analysis of microscopic images, with particular emphasis on deep learning (DL). Second, efforts were made to seek a solution within deep networks with a relatively simple structure. In the paper, we present an approach based on the semantic segmentation of the microscopic images of fuel guesses. The outline of the visual information processing path is as follows: The images are gathered using the camera coupled with an optical microscope. Once the images are gathered, they are preprocessed and then used as an input for the developed deep network. The network is trained to recognize the intrusions expected in the pellets, such as rust, metal, bark, plastic, and so on. The output of the DL is just a kind of image showing the distribution of the intrusions recognized in the input image.

Computer vision is one of the most intensively developing fields of artificial intelligence. It is increasingly used in medicine [22,23,24], energy and the mining sector [25,26,27,28], agriculture [29,30,31], and civil engineering [32,33,34], to name a few. Computer vision methods utilizing deep learning were used for segmentation of the corneal endothelium [22]. The superior results were obtained by following the application of deep learning with postprocessing. It allowed for precise distinction between corneal endothelium cells. The application of texture features was used for caries detection [23]. The analysis of beam computed tomography with computer vision methods allowed the determination of the properties of the temporomandibular joint [24]. Computer vision was used in the monitoring of the mineral enhancement process [25]. Deep learning was used to differentiate between coal and barren rock [27]. Artificial intelligence methods were used for grain boundary segmentation in aggregates [28]. The mentioned methods were also used in plant phenotyping [29], crop estimation [30], and plant condition assessment [31]. There were also many attempts targeting the application of computer vision and artificial intelligence in petrography [20,35].

Microscopic vision, segmentation, and deep learning have been successfully applied to recognize petrographic components and mineral substances in coal [17], Other researchers have used computer vision methods to identify minerals in rocks [20,21] or determine the grain sizes of aggregates [26]. Since the same components (coal, minerals, rocks) are present in wood pellets and are treated as contaminants, attempts have been made to apply similar microscopic and deep learning methods to their recognition.

The core of the work focuses on the interpretation of the microscopic image to identify visible petrographic entities [20]. There are several techniques proposed, such as analysis of reflected light intensity [36,37], color and texture feature analysis [38,39], and application of machine learning methods for turning the proposed feature sets into mineral descriptions.

Deep learning is currently one of most exciting research areas. Deep neural networks were extremely successful in many computer vision applications [40,41]. There have also been very promising attempts at using deep networks for autonomous analysis of microscopic images [18,42]. A UNet [43]-based network was used for maceral group identification in coal sample cross-sections observed with an immersed microscope yielding satisfactory results [17]. Other researchers reported promising results using selected (SegNet [44], DeepLabv3 [45]) deep networks for maceral identification [19].

Despite the many attempts at the application of computer vision and deep learning in petrology and microscopic image analysis, there have been almost no attempts at targeting the identification of intrusions in pellet fuels. The studies in petrography, and particularly coal petrography which is the most similar, used relatively big and complicated networks [18,19]. The simpler networks, such as the feed-forward network, were used only as a classifier applied to the feature vectors calculated using the texture [46,47] or color properties of the image [48]. A different approach is taken in this research. The well-known UNet encoder–decoder architecture is taken as an inspiration and the proposed networks using a number of filters are tested for robustness in the pellet fuel image segmentation. The influence of input image resolution on the segmentation results and learning process is also tested. To the authors’ best knowledge, neither of these approaches has been taken before in the case of immersed microscopic image segmentation.

The problem of intrusions is very important, as the unwanted substances and compounds can be harmful for the environment and human health. The present research addresses this issue. The scope of the work is as follows:

1. Semantic segmentation is used for the identification of intrusions in the microscopic, immersed images of pellet fuels;
2. Research of different UNet-based architectures and their robustness in the intrusion segmentation was performed;
3. The influence of input image resolution on the final segmentation results and best deep network structure was investigated.

The research focused on one of the best-known network architectures: the UNet. During the experiments, its structure (number of filters as well as the depth) was varied to test its influence on the final segmentation results.

## 2. Materials and Methods

### 2.1. The Research Outline

**Figure 1:**
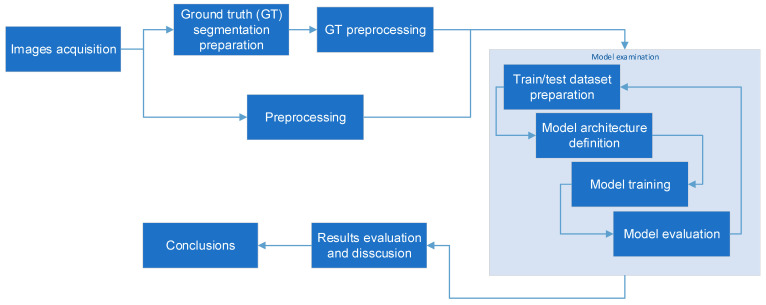
The research outline.

The research targets a few aspects of semantical segmentation of microscopic images of pellet fuels. The overall outline falls within the domain of supervised learning [49,50]. The examined deep network is first trained with the set of image pairs. The image pairs consist of the microscopic image along with its reference segmentation. Then, the quality of the model is assessed using similar image pairs set (testing set) containing images not used for the training phase. The process is repeated using various deep network architectures and training approaches. The training/testing cycle is performed many times, each time changing the training conditions. The obtained models are assessed and analyzed within the perspective of the intended research goals. The overall process of data preparation, model structure assumptions, training, and results assessment is schematically presented in Figure 1.

The detailed descriptions of the outlined steps are provided in the following subsections.

### 2.2. Pellet Sample Preparation and Image Acquisition

The preparation of solid biomass samples for petrographic analysis is similar to that used in coal testing [51]. The solid biomass sample is air-dried [52] well mixed, quartered, and a sample of approximately 0.5 kg is separated. The sample should be thoroughly crushed to a grain size below 1 mm with a minimum amount of fine particles; this is usually less than 1% of the original sample weight. Shredded material, sifted through a 1–0.5 μm sieve, coated with struers SpeciFix-20 epoxy resin, and cured according to [51]. Grinding and polishing should be in accordance with [53] using a Struers LaboForce-3 grinding and polishing machine (Struers Inc., Cleveland, OH, USA). First, the sample was sanded on water sanding paper with a gradation of 800, then 1200, and finally polished on a polishing disc. The polished preparation was washed under running water, then distilled water, and then dried.

The preparation was subjected to microscopic examination with white reflected light at a magnification of 500× in immersion oil (compatible with type F immersion fluid) according to [54] (refractive index *n* = 1.5180). A Zeiss Axio Imager Z 2 m microscope (Carl Zeiss AG, Oberkochen, Germany) was used for the study. Areas were selected for shooting with the Axiocam 506 color camera. One pixel on the resulting image is representative of a 0.091 μm × 0.091 μm square area of the used sample. The resulting set of microscopic images showed different markers for which a set of masks was developed. In the petrographic analysis, the most important was the contribution of the largest group of impurities, which are found in barbecue fuels and wood pellets; the background was epoxy resin.

### 2.3. Ground Truth Image Preparation

**Figure 2:**
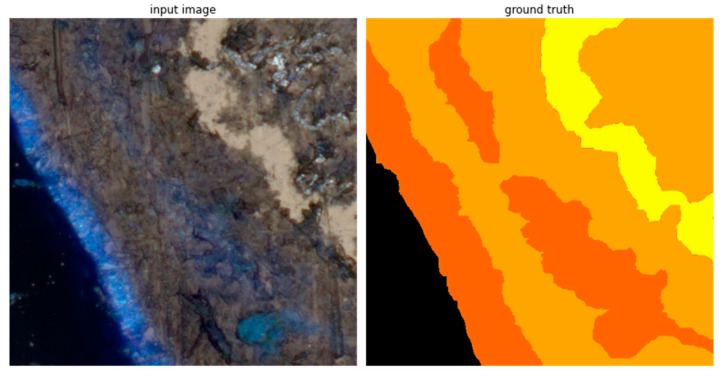
Example of ground truth segmentation. The real size of the presented images is 46.592 μm × 46.592 μm.

Supervised learning uses sets of pairs forming training (and testing) data. Each pair contains an input feature vector (an image in case of semantic segmentation) and a reference-awaited response for the input vector (a reference split into semantic classes). The input data are gathered according to the procedure described in the previous section. The reference segmentations—the ground truth—have to be prepared by domain experts. In case of immersed microscopic images of pellet fuels, the ground truth segmentation was prepared in a few steps. First, the built-in capabilities of the Zeiss Zen Intellisis software were used to obtain a semiautomatic, first approximation of the desired reference. Then, the segmentations were corrected manually by domain experts with help from the well-known GIMP graphic editor. During the manual segmentation, 9 intrusions were identified within the images: coal, rust, metal, biomass, bark, ash, charcoal, plastic and mineral matter. Each intrusion was given a label and was depicted in the ground truth image with a different color. The example of the ground truth segmentation is presented in Figure 2. In Table 1, the assigned labels and their representing colors are presented. The background represents all matter not classified in terms of the 9 intrusions identified.

**Figure 3:**
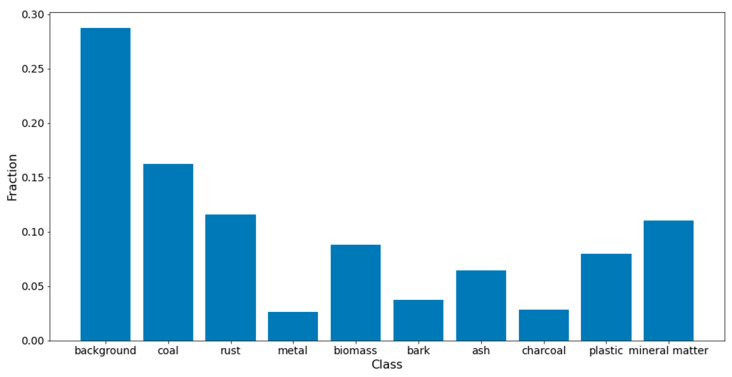
Class histogram.

The obtained ground truth images were used for estimation of possible class imbalances in the input dataset. The nature of the utilized microscopic images suggests that class imbalance is not significant. However, to verify this assumption, a histogram was created representing the fraction of pixels belonging to each class relative to all pixels in the image. This histogram was created cumulatively based on all prepared images. From observations, it can be inferred that class imbalance for metal, bark, and charcoal is noticeable. This problem was addressed by employing loss functions resilient to class imbalance in the training data and using average per-pixel accuracy and average intersection over union (*IoU*) calculated per class as performance measures for the obtained models. The class fraction histogram is presented in Figure 3.

### 2.4. Preprocessing

Before the images can be used in the deep network learning/testing pipeline, they have to be preprocessed. The preprocessing is different for the input images and the ground truth reference segmentations. Each of the preprocessing pipelines is described in the sections below.

#### 2.4.1. Input Image Preprocessing

The input images were 3 channel RGB images. They were spitted into square tiles measuring 1024 × 1024 pixels each. Depending on the test requirements, the images were then rescaled into lower resolutions: 512 × 512 and 256 × 256 pixels. The different sizes were prepared for further investigation of the resolution’s (raster) influence on segmentation results. The images were then augmented to increase variability of the training and testing sets. The augmenting operations were chosen to represent the possible diversity of the obtained microscope images. Therefore, they were limited to vertical/horizontal split translations and rotations with multiples of the right angle. Each transformation of input images was synchronized with the same transformations completed with its ground truth image. The augmentation influences the variety of the training set, thus helping to avoid model overfitting. The last preprocessing operation rescales the color channel range from integer 0–255 into float 0.0–1.0, which is the required input value range for a UNet neural network.

#### 2.4.2. Ground Truth Preprocessing

Ground truth image preprocessing, apart from augmenting the transformations described above, required transforming them into a form suitable for the supervised learning process. It was assumed that each of the identified classes is represented by “one hot” encoding. The background was treated as an additional 10th class. The encoding transformed a 3-channel RGB image into a 10-channel image. Each channel was just a binary image depicting the location of the pixels of the representative class. Such 10 channel images were used both for deep network training and testing.

### 2.5. Network Architecture and Learning Approach

**Figure 4:**
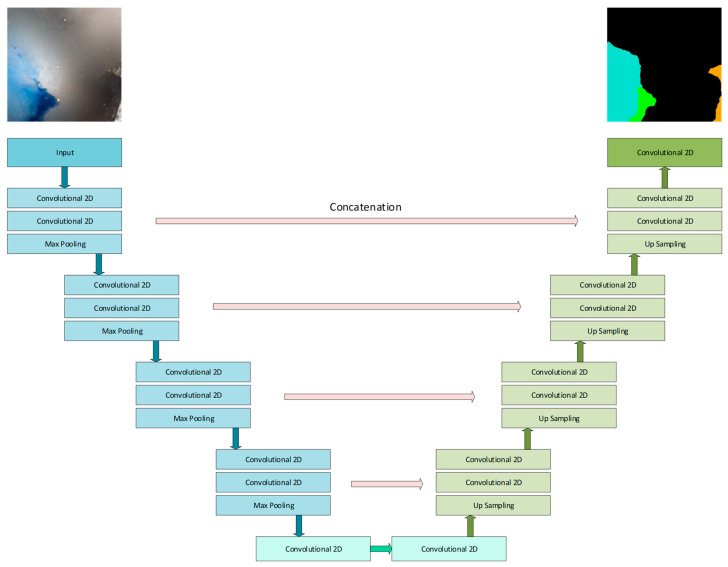
The base UNet architecture used for multiclass pellet fuel segmentation. The architecture was varied during the experiments.

For the segmentation experiments, the well-known UNet architecture-based networks were selected. The network has an encoder–decoder structure, with the skip connections helping in detail reconstruction during the segmentation construction in the decoder part. The network’s typical architecture is depicted in Figure 4. The UNet was chosen because of its relatively simple and flexible structure, easy adaptation to input image size, the robustness reported in previous research, and the wide possibility for modifications (such as adding supplementary dropout and batch normalization layers, adding attention modules, and so on). The network structure was modified during the experiments in the following manner:

- The number of filters at each of encoder/decoder section were changed;
- The depths of the encoder/decoder parts were varied (number of sections composing the encoder and decoder parts).

The model’s performance was assessed using 5-fold cross validation. Each fold was of approximately equal size. The split into folds was performed randomly using the scikit-learn library. Care was taken to ensure that the class representation within the folds corresponded to the representation in the input dataset. The training procedure used input data in two separate parts: a training set containing 80% of all images (4 folds) and a testing set composed with the rest of them (remaining folds). Both sets were shuffled before the start if the training/testing procedures. At each learning cycle, the network was initialized using Glorot initialization [55]. Then, the network was trained for 300 epochs with early stopping conditions (the learning process stops after 10 iterations without loss function improvement). The ADAM [56] optimizer with a learning rate varying from 10^−3^ to 10^−6^ was used for network weight optimization. The categorical cross-entropy was used as a loss function:

(1) $$L_{CE}=-\sum_{c=1}^{n_{C}}y_{c}^{GT}\log\left(y_{c}^{pred}\right),$$

where *L_CE_* is the cross entropy value for one sample; $y_{c}^{GT}$ is a ground truth for class *c*; $y_{c}^{pred}$ is the predicted class *c* probability (taken as the output of the softmax last layer activation function); $n_{C}$ stands for number of classes considered (10 in the described case). The *L_CE_* is summed up for all samples used during the training process to calculate the total categorical cross-entropy loss.

During the computations, the per pixel accuracy, the intersection over union (*IoU*), and the mean *IoU* (*mIoU*) were monitored for both training and validation sets:

(2) $$IoU\left(S_{GT},S_{pred}\right)=\frac{\left|\;S_{GT}\cap S_{pred}\right|}{\left|S_{GT}\cup S_{pred}\right|},$$

where *S_GT_* and *S_pred_* represent the ground truth and predicted pixels sets for a given class. The mean *IoU* is just calculated as an average of the *IoU* for all classes.

Each experiment was carried out using three selected image sizes: 256 × 256, 512 × 512, 1024 × 1024. The results were assessed using the *mIoU* value, treating it as the most important for the quality of the multiclass semantic segmentation. The images were also examined by an experienced domain expert.

## 3. Results

### 3.1. Experiment Setup

All calculation routines were prepared using Python computer programming language with the use of the Jupyter notebook environment [57,58,59]. The developed code extensively used publicly available, open source libraries supporting mathematical, machine learning, and deep learning computations, such as NumPy, Scikit-learn and Tensorflow [60,61,62]. All deep learning models were implemented using Tensorflow. For image preprocessing, a data generator was developed. It was responsible for images and ground truth loading from file; applying appropriate preprocessing; and shuffling and supplying the image/ground truth pairs into the training/testing model procedure.

The computations were performed on the PC workstation running Microsoft Windows 10 (Microsoft Corporation, Redmond, WA, USA) operating system. The computer was equipped with the Intel Core i7-6850K CPU (Intel Corporation, Santa Clara, CA, USA), 32 GB of RAM memory and Nvidia GeForce 1080 (Nvidia Corporation, Santa Clara, CA, USA).

### 3.2. Segmentation Results

The computations were performed in different combinations of the network architecture and image resolutions. The tested network structure ranged from an architecture containing the following numbers of filters in the consecutive encoder and decoder parts:

- Architecture A1: 4, 8, 16, 32, 64 filters;
- Architecture A2: 8, 16, 32, 64, 128 filters;
- Architecture A3: 16, 32, 64, 128, 256 filters.

**Figure 5:**
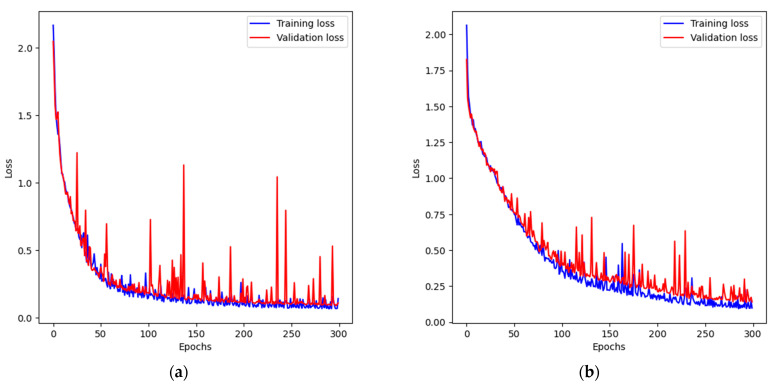

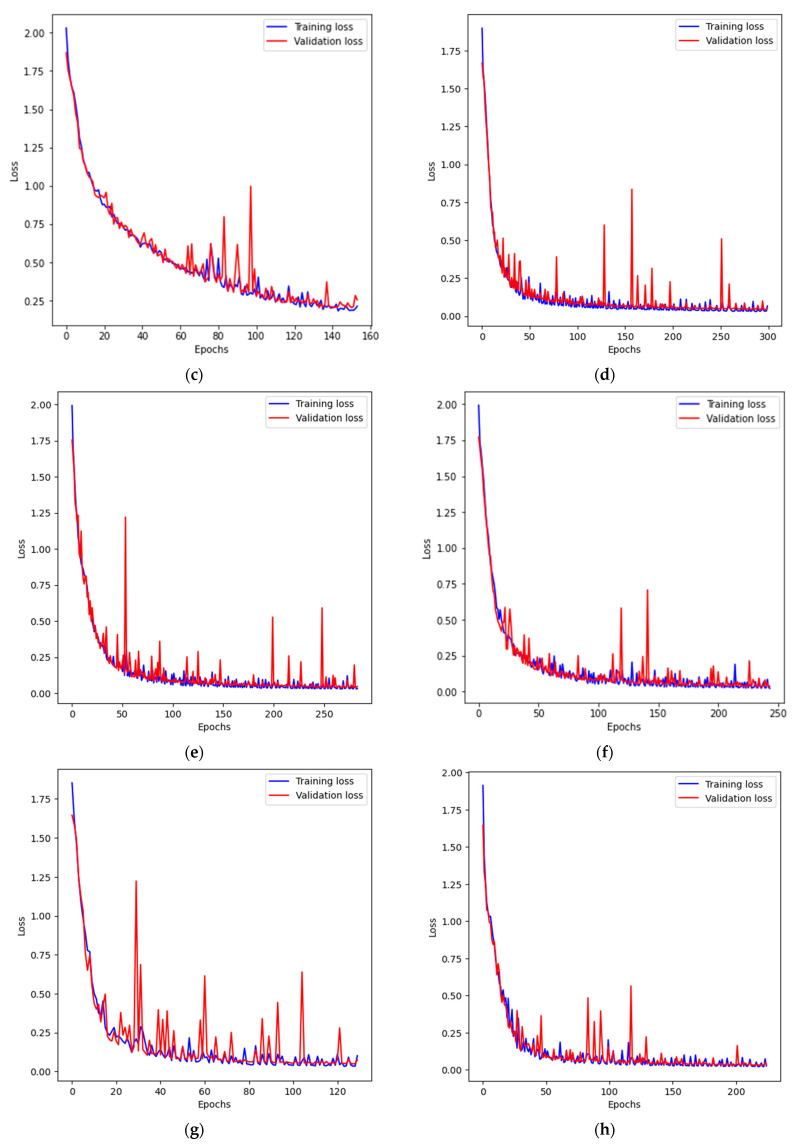

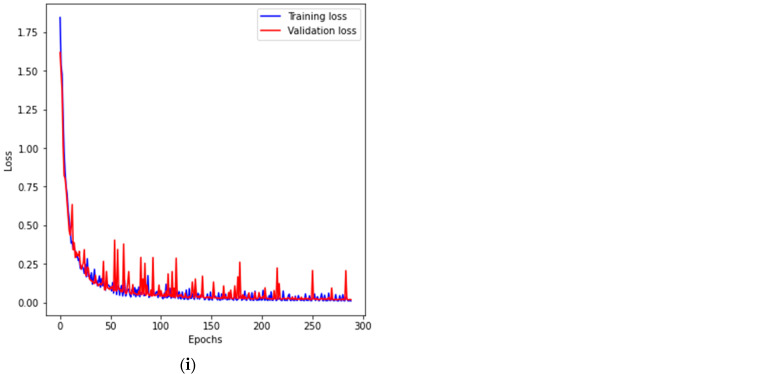
The loss function change during training process: (**a**) A1 network, 256 × 256; (**b**) A1 network, 512 × 512; (**c**) A1 network 1024 × 1024; (**d**) A2 network, 256 × 256; (**e**) A2 network, 512 × 512; (**f**) A2 network, 1024 × 1024; (**g**) A3 network, 256 × 256; (**h**) A3 network, 512 × 512; (**i**) A3 network, 1024 × 1024.

The variations in input image resolution (256 × 256, 512 × 512, 1024 × 1024) allowed testing of the importance of the filters’ actual receptive field size in terms of input image coverage. During the training process, the loss function values for the training and validation sets were recorded to assess the quality of the learning and identify any possible pitfalls (e.g., overfitting). The charts for the tested cases are presented in Figure 5.

## 4. Discussion

The analysis of the presented charts shows that, in the case of the smallest model, the training process was rather difficult. There were many iterations for which the loss function increases rapidly and then decreases. Finally, the model managed to converge, but the resulting measures were the worst from all cases. The A2 and A3 architectures learned smoothly and with satisfactory results.

We attempted to use Zeiss Intellesis software for segmentation, which involves point-wise color marking of the various types of contaminants present in wood pellets. Segmentation entails repeating the process of recognizing contaminants multiple times, but it did not yield satisfactory final results.

**Figure 6:**
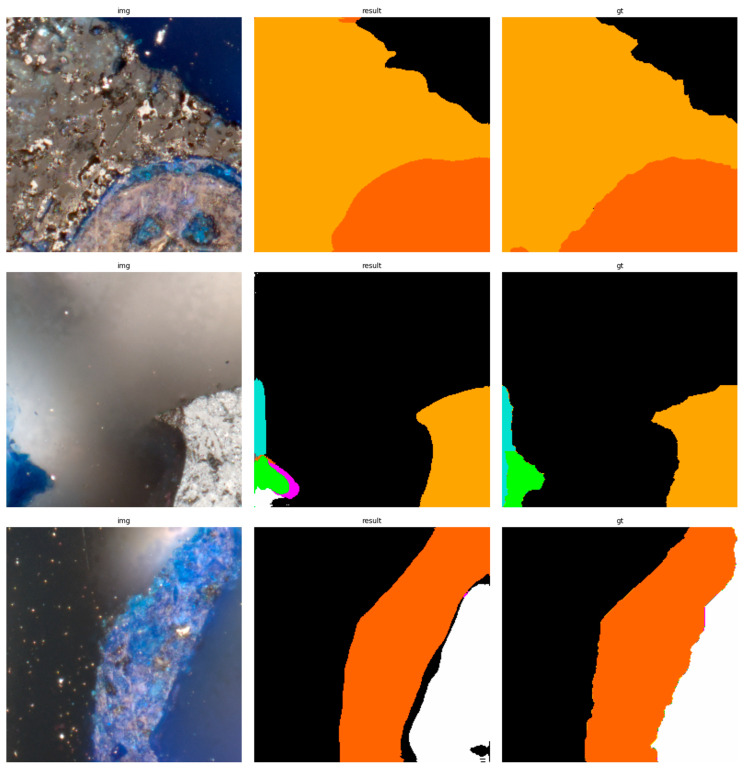
Examples of segmentation results for A1 network, 256 × 256.

**Figure 7:**
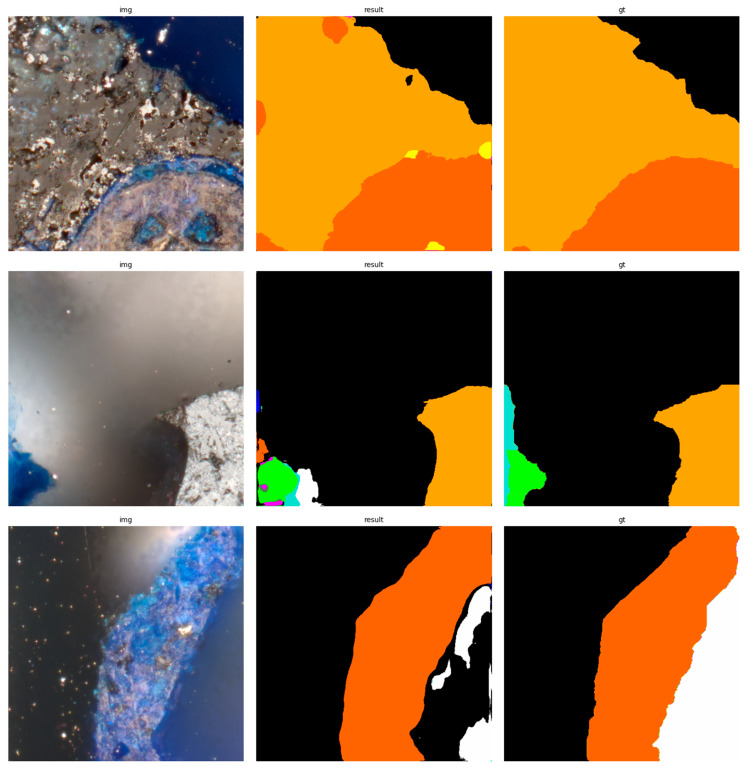
Examples of segmentation results for A1 network, 512 × 512.

**Figure 8:**
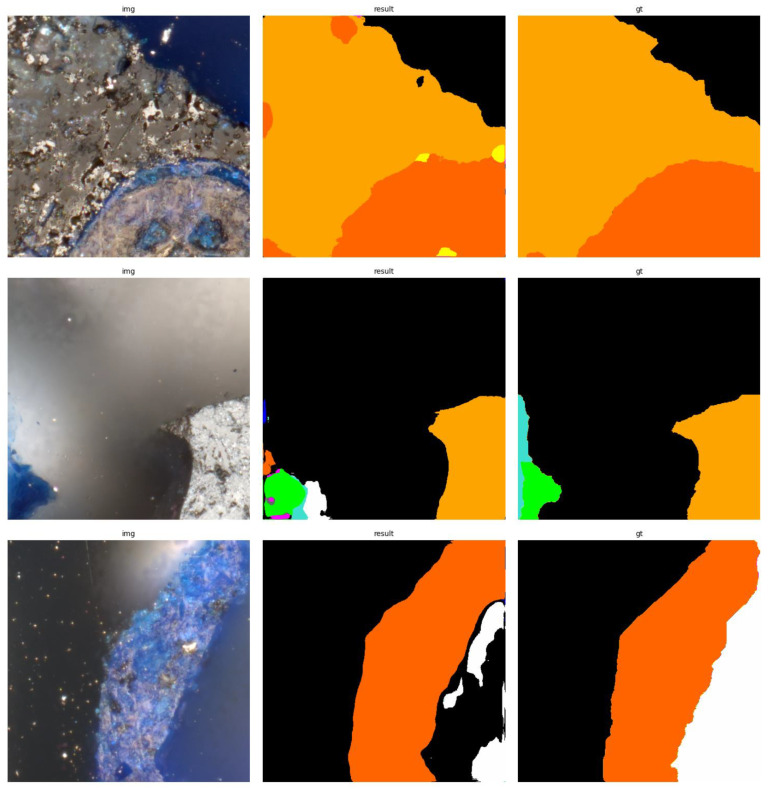
Examples of segmentation results for A1 network, 1024 × 1024.

**Figure 9:**
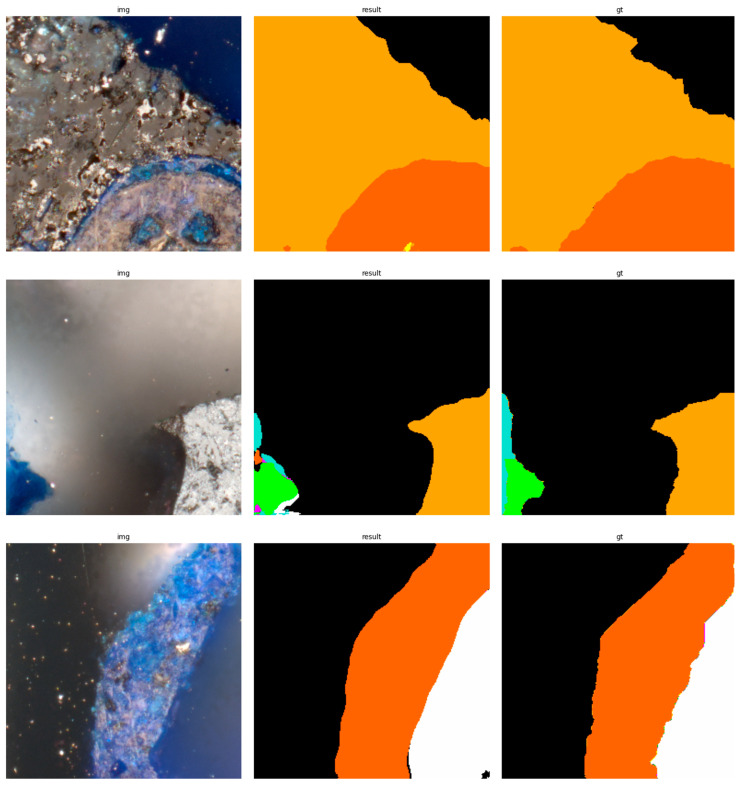
Examples of segmentation results for A2 network, 256 × 256.

**Figure 10:**
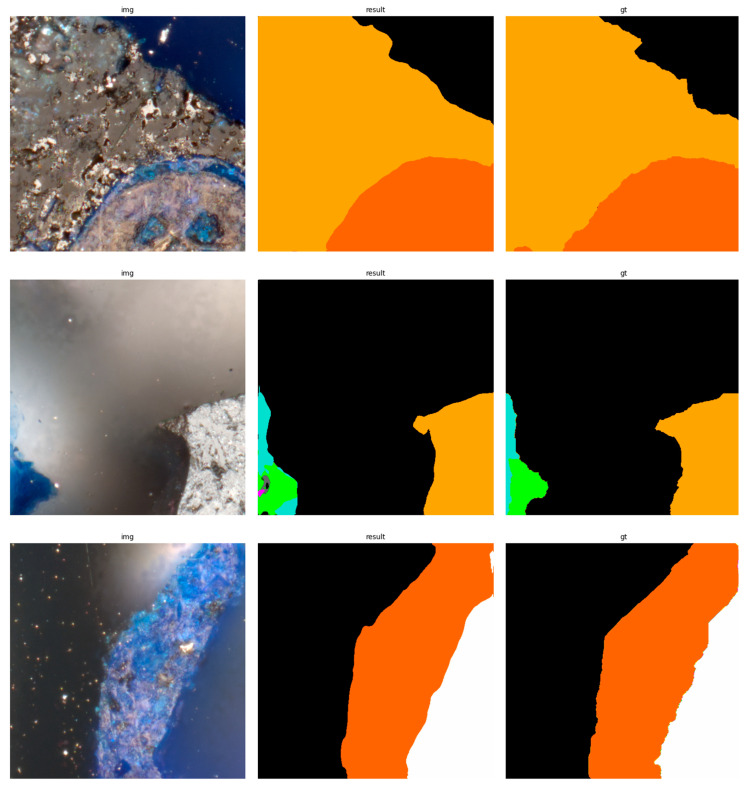
Examples of segmentation results for A2 network, 512 × 512.

**Figure 11:**
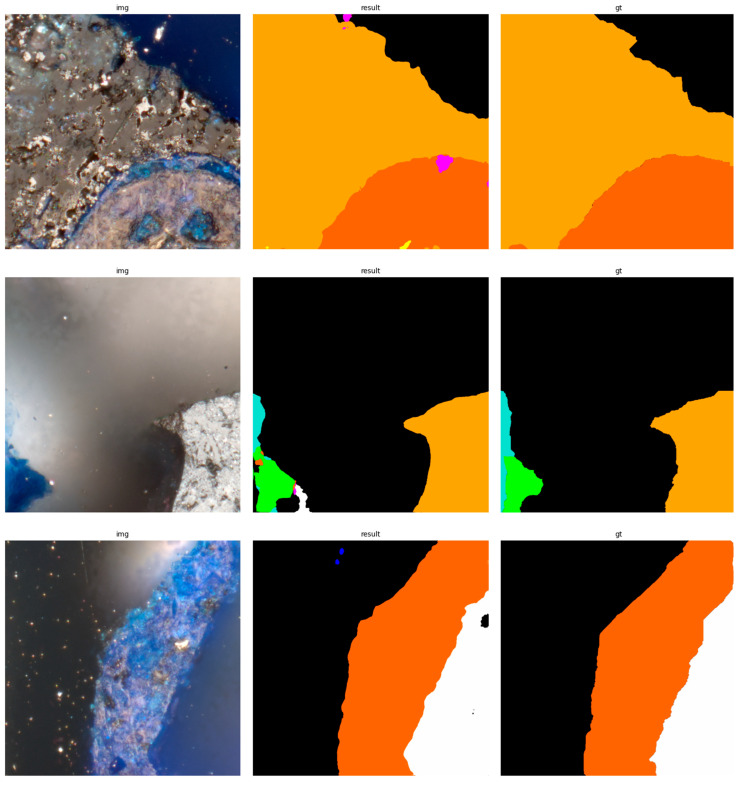
Examples of segmentation results for A2 network, 1024 × 1024.

**Figure 12:**
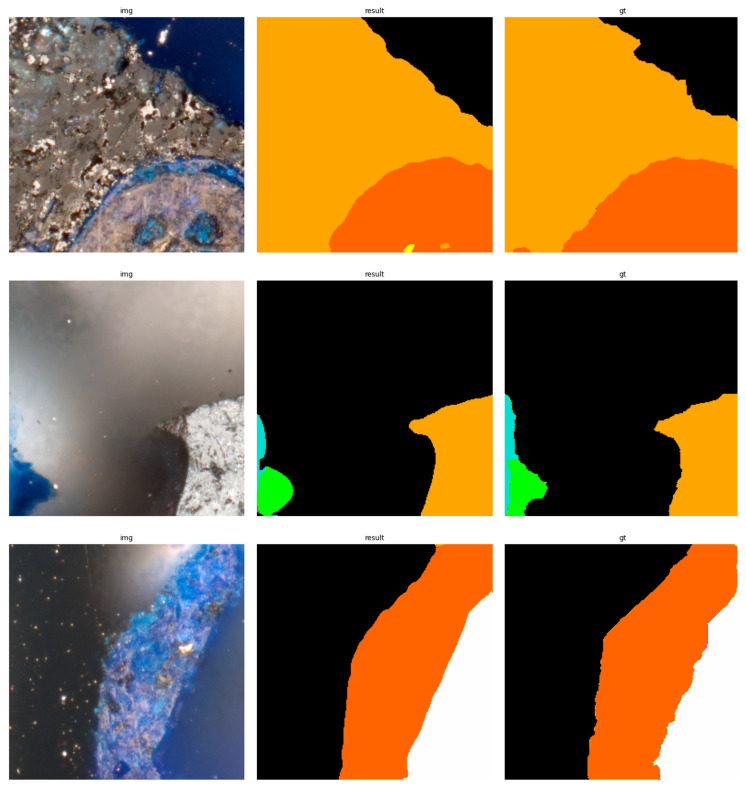
Examples of segmentation results for A3 network, 256 × 256.

**Figure 13:**
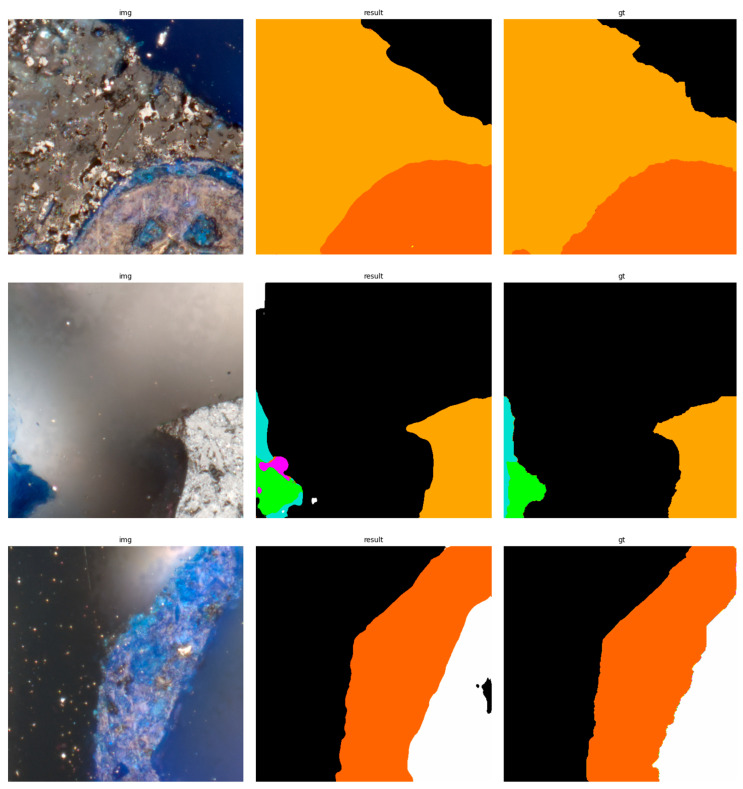
Examples of segmentation results for A3 network, 512 × 512.

**Figure 14:**
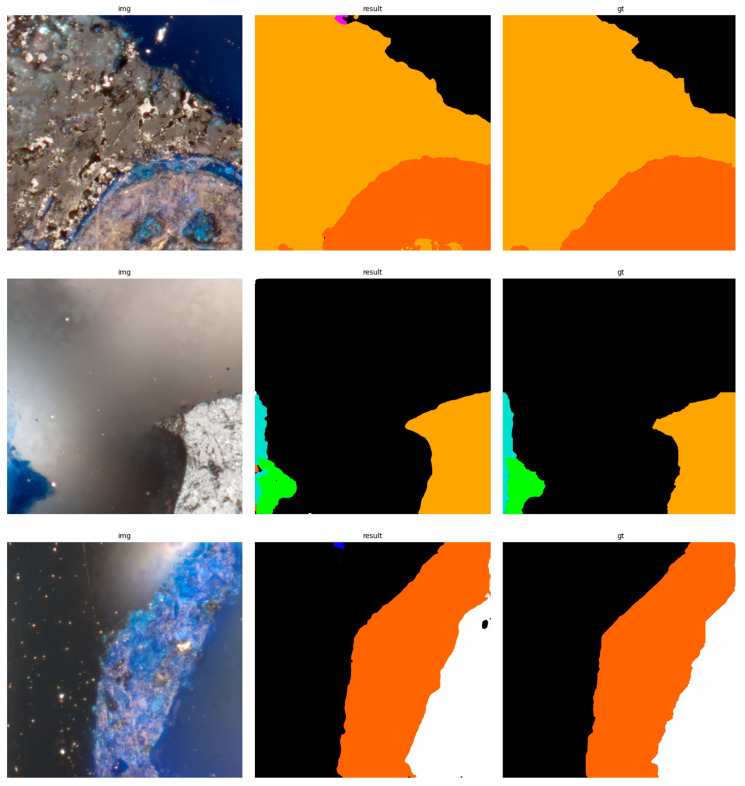
Examples of segmentation results for A3 network, 1024 × 1024.

The results of using the trained models utilized to predict the segmentation for images in the test set are presented in Figure 6, Figure 7, Figure 8, Figure 9, Figure 10, Figure 11, Figure 12, Figure 13 and Figure 14. It is easily observed that increasing the number of filters in the network causes an improvement in segmentation quality. In fact, for the A1 architecture, the number of filters in the first section of the network hardly exceeds the number of channels in the input image. However, the obtained results are interesting: though not precise, the network was able to discover most of the classes. It is also interesting that there is not a lot of difference in segmentation quality between the tested resolutions. All of them produced a segmentation of limited quality, but the images of higher resolution were segmented slightly better. The A2 architecture gave significantly better results. The segmentations, although not free from artefacts, are much better than the ones achieved with the A1 network. Again, in the cases of higher resolutions, the results are significantly better. This is even more true for the A3 architecture. The network was able to accurately segment most of the images. Segmentation errors, such as mistaking the classes or recognizing the intrusion class as a background, are observed in only a few of them. Most of the images which caused the problems for the network were in gray color tones (as if all color channels did not differ enough from each other). It is possible that the limitation in color variability made the appropriate segmentation more difficult.

In the table below (Table 2), the measures of segmentation quality calculated for the test set of images are presented. One can spot that the dependence of the measures on the resolution and depth of the network used is not obvious. When the shallowest network is considered, the increase in image resolution has an adverse effect on segmentation quality. It is true for almost all the measures. The only slight exception is the value of *mIoU* for the 512 × 512 resolution. The values gathered for the A2 architecture do not expose a noticeable or monotonous change when the resolution varies. Finally, the last of the tested architectures, A3, follows the pattern of increasing the measures of segmentation quality in accordance with the increasing resolutions.

The behavior seems to be rather counterintuitive at the first glance. Analyzing these cases, we concluded that the observed phenomenon is related to the possibilities for extracting information (in the form of features that the individual layers of the network learn to extract). The poorest network has only four filters in the first layer. This means that the network has to extract the most important features from the image, with no chance to use the more refined ones. Therefore, if we introduce an image scaled to a lower resolution as input (256 × 256 in the analyzed case), the network can focus on general structures. In a sense, providing an image with higher resolution means that the network, due to the filter size used, must focus on small areas where color variability is related more to the local characteristics of the existing textures, thus reducing the ability of the network to adapt to the correct segmentation of larger areas. Scaling the image to a lower resolution therefore acts as a kind of pre-filtering, removing information that a less extensive network is unable to use. It is interesting that the change in this behavior was observed only for the network with 16 filters in the input layer. Bearing in mind the above analysis, it should be noted, however, that the quality of the segmentation for the A1 architecture is far from perfect. Much better results can be achieved with the A2 architecture. Fully satisfactory results were achieved in the case of the A3 architecture, but it is the most demanding network from the computational point of view. Therefore, it seems reasonable to say that it should be used in practice, although if a rough assessment is enough, the network with A2 architecture is also worth considering. In general, it can be stated that the obtained segmentation results are fully satisfactory, and deep neural networks are a valuable tool supporting the analysis of microscopic images of pellet solid fuels.

To further investigate the effectiveness of the developed models, mean *IoU* was calculated for each contaminant class across the samples (as the opposite to the *mIoU*, where the mean is calculated across the classes). Apart from that, the standard deviation was also calculated for each of the averaged *IoU* values. The bigger the standard deviation is, the potentially less stable the working of the model for the corresponding contaminant class. The results are presented in the Table 3.

Looking at the data presented in the table, it may be concluded that the network achieves the best effectiveness by segmenting the mineral matter, while for metal intrusions the performance is the worst. Meanwhile, plastic is the contaminant for which the network’s performance is the most stable. The network has the biggest confusion in the segmentation of the biomass. Having stated that, it is noticeable that the overall performance for each class is high. The samples used in the determination of the values presented above were taken only from the test set.

Although the proposed A3 architecture is the largest of the nets, it is still much simpler than the one proposed by the basic UNet literature [43]. In fact, although simpler, the A3 and A2 architectures were capable of successfully segmenting microscopic images. To try to improve the performance, one can increase the number of filters and even the depth of the network. However, as a consequence, an increase in computation time and memory consumption is to be expected. Finding a relatively simple network that allows the obtention of good segmentation results is a more tempting approach to follow. The results obtained were compared to the ones achievable using the semiautomatic segmentation functionality of the Zeiss Intellesis software. The software uses deep layers from VGG16/VGG19 [63] networks for feature extraction from an image. Then, the features are used with the random forest (RF) classifier [64]. The user is expected to train the model (RF) several times to obtain the reasonable segmentation. While the approach is recognized as satisfactory for biological materials, it did not provide good results in the case of immersed microscopy imaging of pellet fuels. However, the functionality was of great help during the preparation of ground truth images. In contrast, trained DL models performed well for the test set. The good performance and possibility of simplifying the network structure are also connected with the image acquisition method. It enforces stable lighting and repeatable conditions, making the segmentation of newly acquired images easier. Looking forward to ways of increasing of segmentation effectiveness, the application of different network architectures might be considered, like DeepLab [45] or SegNet [44]. The attention mechanism is also worth testing in future research [65]. Another prospective field of research is an application of the formal method for deep learning model analysis as described in [66,67].

The application of optical microscopy to the examination of the quality of wood pellets, which will soon become mandatory for their manufacture, is becoming very important. The use of machine learning techniques in this field will streamline the testing procedure. In the future, these investigations will need to encompass all contaminants present in wood pellets. The research methods developed for wood pellets used in grilling can be transferred to the examination of solid biofuels used in thermal power generation.

Microscopic examinations of contaminants in wood pellets conducted by humans are subject to significant error and depend on the knowledge possessed by the operator identifying the contamination in the microscopic image. Another challenge is preparing a proper microscope slide that enables achieving good sharpness under the microscope. The operator faces difficulty in recognizing the boundaries of grain-like contaminants, which are often blurred. The presence of this blurring poses a significant challenge in the process of segmentation and learning, requiring the development of additional methods to address such issues.

## 5. Conclusions

UNet architecture-based networks were used for segmenting intrusions visible in images taken using an optic microscope immersed in an oil layer. The research focused on estimating the influence of network depth (considered as the number of filters used in each convolutional filter block) and image resolution on the gained result. Upon research, the following conclusions were formulated:

- The UNet-based architecture is capable of achieving excellent segmentation results in the case of microscopic images. The quality is sufficient for automating the process and is helpful for domain experts in their daily work;
- The network depth has a big influence on achieving results. The deeper the network, the better results observed. However, it comes with the calculation of costs. It seems reasonable to use networks of at least an A2 structure for most of practical applications. The A3 structure is more than sufficient for automating the segmentation process;
- Increasing the resolution helped in gaining better segmentation results, but only if the network was deep enough. The difference between resolutions for the deepest tested networks is moderate. For practical applications, the 256 × 256 resolution is a good choice as a tradeoff of resources in consumption vs. segmentation results;
- The research results obtained indicate that there are still many areas for further investigation in the subsequent stages. These include, among others, refining the technique of capturing photographs to achieve better sharpness across the entire surface of the analyzed contamination. Further studies will focus on other contaminants present in wood pellets that have not been considered in the previous analyses. The research may also encompass those contaminants for which poor results were obtained;
- The application of the presented models in petrographic expert practice should help lower the effort connected to contaminant identification in pellet fuels.

## Figures and Tables

**Table 1:** The identified classes along with assigned label and color.

Class Name	Label	Color
Background	0	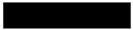
Coal	1	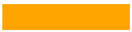
Rust	2	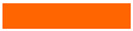
Metal	3	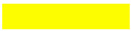
Biomass	4	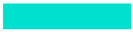
Bark	5	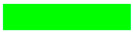
Ash	6	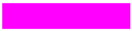
Charcoal	7	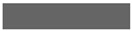
Plastic	8	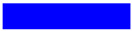
Mineral matter	9	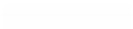

**Table 2:** The per pixel accuracy and the *IoU* measure for tested models and resolutions (all results for test images set).

DL Network	Resolution	Accuracy	*IoU*	*mIoU*
A1	256	0.9586	0.9147	0.5373
A1	512	0.9573	0.9083	0.4312
A1	1024	0.9159	0.8778	0.4063
A2	256	0.9773	0.9504	0.6189
A2	512	0.9814	0.9429	0.5839
A2	1024	0.9823	0.9528	0.5799
A3	256	0.9763	0.9398	0.5854
A3	512	0.9876	0.9597	0.71988
A3	1024	0.9913	0.9799	0.7489

**Table 3:** The *IoU* values averaged across the samples for each class and corresponding standard deviation (A3 architecture, 1024 × 1024 resolution).

Class Name	*IoU* (Averaged)	Standard Deviation
Background	0.9663	0.04695
Coal	0.8911	0.20052
Rust	0.9404	0.05283
Metal	0.7365	0.21255
Biomass	0.8712	0.23070
Bark	0.9688	0.03568
Ash	0.9576	0.04225
Charcoal	0.9543	0.02321
Plastic	0.9649	0.01265
Mineral matter	0.9823	0.02276

## Data Availability

Data is unavailable due to privacy restrictions.

## References

[1] Boman C., Nordin A., Boström D., Öhman M. (2004). Characterization of Inorganic Particulate Matter from Residential Combustion of Pelletized Biomass Fuels. Energy Fuels.

[2] Naeher L.P., Brauer M., Lipsett M., Zelikoff J.T., Simpson C.D., Koenig J.Q., Smith K.R. (2007). Woodsmoke Health Effects: A Review. Inhal. Toxicol..

[3] Yang C.-C., Jenq S.N., Lee H. (1998). Characterization of the Carcinogen 2-Amino-3, 8-Dimethylimidazo [4, 5-f] Quinoxaline in Cooking Aerosols under Domestic Conditions. Carcinogenesis.

[4] Viegas O., Novo P., Pinto E., Pinho O., Ferreira I. (2012). Effect of Charcoal Types and Grilling Conditions on Formation of Heterocyclic Aromatic Amines (HAs) and Polycyclic Aromatic Hydrocarbons (PAHs) in Grilled Muscle Foods. Food Chem. Toxicol..

[5] Chen B.H., Chen Y.C. (2001). Formation of Polycyclic Aromatic Hydrocarbons in the Smoke from Heated Model Lipids and Food Lipids. J. Agric. Food Chem..

[6] Dyremark A., Westerholm R., Övervik E., Gustavsson J.-Å. (1995). Polycyclic Aromatic Hydrocarbon (PAH) Emissions from Charcoal Grilling. Atmos. Environ..

[7] Kafouris D., Koukkidou A., Christou E., Hadjigeorgiou M., Yiannopoulos S. (2020). Determination of Polycyclic Aromatic Hydrocarbons in Traditionally Smoked Meat Products and Charcoal Grilled Meat in Cyprus. Meat Sci..

[8] Kim Oanh N.T., Nghiem L.H., Phyu Y.L. (2002). Emission of Polycyclic Aromatic Hydrocarbons, Toxicity, and Mutagenicity from Domestic Cooking Using Sawdust Briquettes, Wood, and Kerosene. Environ. Sci. Technol..

[9] Chandrasekaran S.R., Hopke P.K., Rector L., Allen G., Lin L. (2012). Chemical Composition of Wood Chips and Wood Pellets. Energy Fuels.

[10] Jelonek Z., Drobniak A., Mastalerz M., Jelonek I. (2020). Environmental Implications of the Quality of Charcoal Briquettes and Lump Charcoal Used for Grilling. Sci. Total Environ..

[11] Miranda T., Montero I., Sepúlveda F.J., Arranz J.I., Rojas C.V., Nogales S. (2015). A Review of Pellets from Different Sources. Materials.

[12] Jelonek Z., Drobniak A., Mastalerz M., Jelonek I. (2020). Assessing Pellet Fuels Quality: A Novel Application for Reflected Light Microscopy. Int. J. Coal Geol..

[13] Jelonek Z., Drobniak A., Mastalerz M., Jelonek I. Environmental and Human Health Implications of Grilling with Wood Pellets and Chips: Atmospheric Environment X. https://www.biomass.edu.pl/post/the-past-year-in-research-at-thomas-hill-research-center.

[14] EN 1860-2:2005. Appliances, Solid Fuels and Firelighters for Barbecueing—Part 2: Barbecue Charcoal and Barbecue Charcoal Briquettes—Requirements and Test Methods. https://standards.iteh.ai/catalog/standards/cen/61708517-6bd7-490a-8beb-d6ff8c50e72c/en-1860-2-2005.

[15] Duca D., Riva G., Pedretti E.F., Toscano G. (2014). Wood Pellet Quality with Respect to EN 14961-2 Standard and Certifications. Fuel.

[16] Drobniak A., Jelonek I., Jelonek Z., Mastalerz M. (2022). Developing Methodology for Petrographic Analysis of Solid Biomass in Reflected Light. Int. J. Coal Geol..

[17] Iwaszenko S., Róg L. (2021). Application of Deep Learning in Petrographic Coal Images Segmentation. Minerals.

[18] Lei M., Rao Z., Wang H., Chen Y., Zou L., Yu H. (2021). Maceral Groups Analysis of Coal Based on Semantic Segmentation of Photomicrographs via the Improved U-Net. Fuel.

[19] Wang Y., Bai X., Wu L., Zhang Y., Qu S. (2022). Identification of Maceral Groups in Chinese Bituminous Coals Based on Semantic Segmentation Models. Fuel.

[20] Pires de Lima R., Duarte D. (2021). Pretraining Convolutional Neural Networks for Mudstone Petrographic Thin-Section Image Classification. Geosciences.

[21] de Lima R.P., Bonar A., Coronado D.D., Marfurt K., Nicholson C. (2019). Deep Convolutional Neural Networks as a Geological Image Classification Tool. Sediment. Rec..

[22] Nurzynska K. (2018). Deep Learning as a Tool for Automatic Segmentation of Corneal Endothelium Images. Symmetry.

[23] Obuchowicz R., Nurzynska K., Obuchowicz B., Urbanik A., Piórkowski A. (2020). Caries Detection Enhancement Using Texture Feature Maps of Intraoral Radiographs. Oral Radiol..

[24] Iwaszenko S., Munk J., Baron S., Smoliński A. (2021). New Method for Analysis of the Temporomandibular Joint Using Cone Beam Computed Tomography. Sensors.

[25] Kistner M., Jemwa G.T., Aldrich C. (2013). Monitoring of Mineral Processing Systems by Using Textural Image Analysis. Miner. Eng..

[26] Yaghoobi H., Mansouri H., Farsangi M.A.E., Nezamabadi-Pour H. (2019). Determining the Fragmented Rock Size Distribution Using Textural Feature Extraction of Images. Powder Technol..

[27] Pu Y., Apel D.B., Szmigiel A., Chen J. (2019). Image Recognition of Coal and Coal Gangue Using a Convolutional Neural Network and Transfer Learning. Energies.

[28] Iwaszenko S., Nurzynska K., Iwaszenko S. (2020). Application of Texture Features and Machine Learning Methods to Grains Segmentation in Rock Material Images. Image Anal. Stereol..

[29] Chandra A.L., Desai S.V., Guo W., Balasubramanian V.N. (2020). Computer Vision with Deep Learning for Plant Phenotyping in Agriculture: A Survey. arXiv.

[30] Zheng Y.-Y., Kong J.-L., Jin X.-B., Wang X.-Y., Su T.-L., Zuo M. (2019). CropDeep: The Crop Vision Dataset for Deep-Learning-Based Classification and Detection in Precision Agriculture. Sensors.

[31] Kamilaris A., Prenafeta-Boldú F.X. (2018). Deep Learning in Agriculture: A Survey. Comput. Electron. Agric..

[32] Zhang J., Zi L., Hou Y., Wang M., Jiang W., Deng D. (2020). A Deep Learning-Based Approach to Enable Action Recognition for Construction Equipment. Adv. Civ. Eng..

[33] Katsamenis I., Protopapadakis E., Doulamis A., Doulamis N., Voulodimos A. (2020). Pixel-Level Corrosion Detection on Metal Constructions by Fusion of Deep Learning Semantic and Contour Segmentation. Proceedings of the Advances in Visual Computing: 15th International Symposium, ISVC 2020.

[34] Huang Y., Fu J. (2019). Review on Application of Artificial Intelligence in Civil Engineering. Comput. Model. Eng. Sci..

[35] Koeshidayatullah A., Morsilli M., Lehrmann D.J., Al-Ramadan K., Payne J.L. (2020). Fully Automated Carbonate Petrography Using Deep Convolutional Neural Networks. Mar. Pet. Geol..

[36] Oestreich J.M., Tolley W.K., Rice D.A. (1995). The Development of a Color Sensor System to Measure Mineral Compositions. Miner. Eng..

[37] O’Brien G., Jenkins B., Esterle J., Beath H. (2003). Coal Characterisation by Automated Coal Petrography. Fuel.

[38] Singh V., Rao S.M. (2005). Application of Image Processing and Radial Basis Neural Network Techniques for Ore Sorting and Ore Classification. Miner. Eng..

[39] Tessier J., Duchesne C., Bartolacci G. (2007). A Machine Vision Approach to On-Line Estimation of Run-of-Mine Ore Composition on Conveyor Belts. Miner. Eng..

[40] LeCun Y., Bengio Y., Hinton G. (2015). Deep Learning. Nature.

[41] Goodfellow I., Bengio Y., Courville A. (2016). Deep Learning.

[42] Li D., Zhang Z., Xu Z., Xu L., Meng G., Li Z., Chen S. (2019). An Image-Based Hierarchical Deep Learning Framework for Coal and Gangue Detection. IEEE Access.

[43] Ronneberger O., Fischer P., Brox T. (2015). U-Net: Convolutional Networks for Biomedical Image Segmentation. Proceedings of the International Conference on Medical Image Computing and Computer-Assisted Intervention.

[44] Badrinarayanan V., Kendall A., Cipolla R. (2017). Segnet: A Deep Convolutional Encoder-Decoder Architecture for Image Segmentation. IEEE Trans. Pattern Anal. Mach. Intell..

[45] Chen L.-C., Papandreou G., Schroff F., Adam H. (2017). Rethinking Atrous Convolution for Semantic Image Segmentation. arXiv.

[46] Haralick R.M., Shanmugam K., Dinstein I.H. (1973). Textural Features for Image Classification. IEEE Trans. Syst. Man Cybern..

[47] Ojala T., Valkealahti K., Oja E., Pietikäinen M. (2001). Texture Discrimination with Multidimensional Distributions of Signed Gray-Level Differences. Pattern Recognit..

[48] Mlynarczuk M., Skiba M. (2017). The Application of Artificial Intelligence for the Identification of the Maceral Groups and Mineral Components of Coal. Comput. Geosci..

[49] Bishop C.M., Nasrabadi N.M. (2006). Pattern Recognition and Machine Learning.

[50] Trevor H., Robert T., Jerome F. (2009). The Elements of Statistical Learning: Data Mining, Inference, and Prediction.

[51] ISO 7404-2:2009. Methods for the Petrographic Analysis of Coals—Part 2: Methods of Preparing Coal Samples. https://www.iso.org/standard/42798.html.

[52] ISO 14780:2017. Solid Biofuels—Sample Preparation. https://www.iso.org/standard/66480.html.

[53] ISO 6344-3:2013. Coated Abrasives Grain Size Analysis—Part 3: Determination of Grain Size Distribution of Microgrits P240 to P2500. https://www.iso.org/standard/56010.html.

[54] ISO 8036:2015. Microscopes—Immersion Liquids for Light Microscopy. https://www.iso.org/standard/67551.html.

[55] Glorot X., Bengio Y. Understanding the Difficulty of Training Deep Feedforward Neural Networks. Proceedings of the Thirteenth International Conference on Artificial Intelligence and Statistics; JMLR Workshop and Conference Proceedings.

[56] Kingma D.P., Ba J. (2017). Adam: A Method for Stochastic Optimization. arXiv.

[57] Millman K.J., Aivazis M. (2011). Python for Scientists and Engineers. Comput. Sci. Eng..

[58] Fangohr H., Kluyver T., DiPierro M. (2021). Jupyter in Computational Science. Comput. Sci. Eng..

[59] Mendez K.M., Pritchard L., Reinke S.N., Broadhurst D.I. (2019). Toward Collaborative Open Data Science in Metabolomics Using Jupyter Notebooks and Cloud Computing. Metabolomics.

[60] Harris C.R., Millman K.J., van der Walt S.J., Gommers R., Virtanen P., Cournapeau D., Wieser E., Taylor J., Berg S., Smith N.J. (2020). Array Programming with NumPy. Nature.

[61] Pedregosa F., Varoquaux G., Gramfort A., Michel V., Thirion B., Grisel O., Blondel M., Prettenhofer P., Weiss R., Dubourg V. (2011). Scikit-Learn: Machine Learning in Python. J. Mach. Learn. Res..

[62] Abadi M., Agarwal A., Barham P., Brevdo E., Chen Z., Citro C., Corrado G.S., Davis A., Dean J., Devin M. (2015). TensorFlow: Large-Scale Machine Learning on Heterogeneous Systems. arXiv.

[63] Simonyan K., Zisserman A. (2014). Very Deep Convolutional Networks for Large-Scale Image Recognition. arXiv.

[64] Breiman L. (2001). Random Forests. Mach. Learn..

[65] Zhou Z., Rahman Siddiquee M.M., Tajbakhsh N., Liang J. (2018). Unet++: A Nested u-Net Architecture for Medical Image Segmentation. Proceedings of the Deep Learning in Medical Image Analysis and Multimodal Learning for Clinical Decision Support: 4th International Workshop, DLMIA 2018, and 8th International Workshop, ML-CDS 2018, Held in Conjunction with MICCAI 2018.

[66] Krichen M., Mihoub A., Alzahrani M.Y., Adoni W.Y.H., Nahhal T. Are Formal Methods Applicable to Machine Learning And Artificial Intelligence?. Proceedings of the 2022 2nd International Conference of Smart Systems and Emerging Technologies (SMARTTECH).

[67] Raman R., Gupta N., Jeppu Y. (2023). Framework for Formal Verification of Machine Learning Based Complex System-of-Systems. Insight.

